# The Royal Army Medical Corps

**Published:** 1919-06-28

**Authors:** 


### THE WORST WAR OF THE WORLD.

### X. THE ROYAL ARMY MEDICAL CORPS.

**"Greater love hath no man than this, that a man lay down his life for his friends."**

**"Be thou faithful unto death, and I will give thee a crown of life."**

What will probably prove to be the last of the Memorial Services in England arising out of this war was held in St. Paul's Cathedral, on 25th inst., in memory of all ranks of the Royal Army Medical Corps who gave their lives in the service of their King and Country in the war of 1914-18. Be this as it may, from the circumstances that all ranks of this splendid corps have had throughout their service mainly to do with the wounded and the sick, the presence in St. Paul's Cathedral of such a large number of members not unnaturally gave the Cathedral an atmosphere which was probably, if not entirely, unique in its history. Our Generals and fighting men throughout the war have properly insisted that the dangers faced and the risks run by the members of the R.A.M.C. in the performance of the duty of all ranks were as great, if not greater than those of the fighting men proper. In the course of their duty, during the active stages of the war, surgeons, matrons, sisters, nurses, orderlies, and all ranks were for many continuous days together constantly associated with terrible suffering and appalling difficulties in treatment. Nursing duties were of the most exacting and prolonged character, and when battles were being fought, the hours of duty were so arduous and continuous, and the strain upon surgeons, matrons, nurses and all ranks of assistants at times so onerous and prolonged, as to make them well-nigh beyond human endurance. Devoted service of this type, being constantly in the presence of death, and almost worse than death, the witness of awful suffering in the case of individuals and the call which it made upon all associated with the patients, must have made the Memorial Service at St. Paul's on 25th inst. recall memories and awaken remembrances of events and superhuman efforts to relieve suffering, which gave to the service a very special and real significance to the majority of those present.

The meaning of what we here point out may be brought more closely home to our readers by recalling to remembrance the Etaples Hospitals on the Western Front, as an example, under the Red Cross flag of protection. This protection, it will be remembered, the Germans disregarded, to their everlasting shame, although it was sacred ground for all other fighting men to whatever nation they might belong, who possess any manliness, courage, or self-respect, let alone feelings of humanity, and the recollection of the claims which the sick and injured have on all hale members of mankind in war or in peace. It was our privilege to spend during the war a considerable time in Etaples, where we made ourselves familiar with the nature and character of the work, the condition and arrangements of the buildings, and the character of the various types of hospitals, the majority of which we personally inspected. We were struck by the fact that there was no attempt to make any clear division between any hospital and its neighbours. The space between each hospital site was simply a space of ground, seldom more than two feet in width, if so much, and it was no uncommon experience for all types of people engaged in the hospitals to lose themselves in trying to make for an adjacent hospital for the first time. There were in this hospital city 26,000 beds, when we inspected it in 1917, and that number had been largely increased by May 19, 1918, when two squadrons of German Gothas flew over it on Whit-Sunday night soon after 10 P.M. and continued their hellish work until midnight. The *Times* reported that over a score of machines in two separate parties were employed, which dropped a great number of bombs, many of them large in size, making craters in the ground 15 and 20 feet across. It would be difficult to imagine any deliberately planned horror, invented by Satan himself, which could be more utterly devoid of any quality which civilised men, and even the most barbarous savages, have recognised should characterise the acts of any member of the human family, where sick, helpless, and dying men and defenceless men and women are concerned. The patients' quarters consisted of huts and tents, slight in character, with the absence of every kind of protection and defence or shelter for their inmates. They contained the patients' beds, which were filled with helpless wounded. Some of them inside presented the appearance of a forest of trees, with the patients with their fractures accurately adjusted and fixed in frames of aluminium, suspended in the air by a system of pulleys, in such a manner that they could move their bodies without disturbance of the injured limbs, the open wounds of which were at all times freely accessible. These cases had to be dressed, too, with infinite devotion and care, in the position in which they might be suspended. Not a few of those present in St. Paul's Cathedral no doubt had in their minds the picture of the inside of one of these hospital huts or tents, vividly recalled to their memories, and it would be well if every man, woman and child throughout the civilised world could have that picture placed before them. But to those members of the congregation who, like ourselves, had in mind another picture showing the German airmen in the Gothas first exhausting their stock of heavy bombs, which they dropped so that they might land somewhere amongst the attendants' quarters, and the tents where the nursing sisters were moving among the rows of beds with their helpless patients, and the later picture when the enemy brought down their infernal machines low enough to enable them, fiends that they were, to use their machine guns direct on the hospitals and

☛ **Previous articles appeared on July 28, Aug. 11 and 18, Sept. 1 and Dec. 29, 1917, Aug. 10, Nov. 16 and Dec. 28, 1918; and June 21, 1919, page 287.**

**The Worst War of the World**—*(continued)*.

attendants' quarters, naturally gave to all of us tender, sobering memories of some, who had there given their lives in the service of their King and Country, on Whit-Sunday evening, 1918. The mortality and terrible injuries already inflicted on the sufferers in these tent and hut hospitals and upon the nursing sisters, orderlies, and doctors, by machine-guns, so deliberately aimed at such close quarters, can be better imagined than described. The vivid recalling of the result, being, as the perpetrators of this deed, their masters and the superior officers, desired it should be, a holocaust of slaughtered humanity, exhibiting the cowardice and concentrated abomination of criminal actions and intent which, prior to its perpetration, had never been attempted, much less exceeded, in the whole history of the world, must have had added to it by this Memorial Service a stirring reality that can never be forgotten.

We have thought it well to give this word-picture so as to enable our readers tc understand and realise how unique in many respects must have been the atmosphere of St. Paul's Cathedral for those who have taken an active part in the war at the Front. If further evidence were needed the Order of Service and its arrangement gave this atmosphere, and the circumstances attending it, full expression and point. It begins with the reminder, "Greater love hath no man than this, that a man lay down his life for his friends." It proceeds:

**"Blow out, you bugles, over the rich Dead,**
**There's none of these so lonely and poor of old,**
**But, dying, has made us rarer gifts than gold."**

Then follow the numbers of officers, warrant-officers, non-commissioned officers and men who have been killed in action or who have died cf wounds or disease in the war of 1914-18. We give the places where they fell, and recall to remembrance the fact that the whole of these valued men offered up their lives, though non-combatants, in the service of their King and Country. The deaths of officers and other ranks total no fewer than 568 officers and 4,634 other ranks, of whom 3,526 officers and other ranks were killed in action or died of wounds, and 1,676 died of disease.

The places where they fell were:

| | Officers. | Other Ranks. |
|---|---|---|
| France ... ... ... ... | 432 | 3,754 |
| Italy ... ... ... ... | 1 | 23 |
| Dardanelles ... ... ... | 23 | 95 |
| Macedonia ... ... ... | 19 | 164 |
| Egypt and Palestine ... | 36 | 462 |
| Mesopotamia ... ... | 48 | 95 |
| East Africa ... ... ... | 9 | 41 |

A glorious record cf brave men, living and acting every one of them as "the Good Samaritan" throughout the war, adding to that glorious service the death sacrifice in the discharge of their duties for their fellows, and in the service of their King and Country.

### The Order of the Service commenced with the hymn,

"The Son of God goes forth to war,
A kingly crown to gain;"

Then followed "I am the Resurrection and the Life, saith the Lord"; "I know that my Redeemer liveth, and that He shall stand at the latter day upon the earth." "We brought nothing into this world, and it is certain we can carry nothing out. The Lord gave, and the Lord hath taken away; blessed be the Name of the Lord."

Then was sung by the choir of St. Paul's Cathedral Psalm xxiii., led by a boy's voice of surpassing excellence and tender feeling: "The Lord is my shepherd: therefore can I lack nothing. He shall feed me in a green pasture; and lead me forth beside the waters of comfort." Then the Lesson, Revelation vi., ninth to seventeenth verse, which begins:

"After this I beheld, and, lo, a great multitude, which no man could number, of all nations, and kindreds, and people, and tongues, stood before the throne, and before the Lamb, clothed with white robes, and palms in their hands;

"And cried with a loud voice, saying, Salvation to our God which sitteth upon the throne, and unto the Lamb.

"And all the angels stood round about the throne, and about the elders and the four beasts, and fell before the throne on their faces, and worshipped God.

"Saying, Amen: Blessing, and glory, and wisdom, and thanksgiving, and honour, and power, and might, be unto our God for ever and ever. Amen.

"These are they which came out of great tribulation, and have washed their robes, and made them white in the blood of the Lamb.

"Therefore are they before the throne of God, and serve him day and night in His temple; and He that sitteth on the throne shall dwell among them.

"They shall hunger no more, neither thirst any more; neither shall the sun light on them, nor any heat.

"For the Lamb which is in the midst of the throne shall feed them, and shall lead them unto living fountains of waters; and God shall wipe away all tears from their eyes."

Then was read The Memorial of the Dead: "Let us remember with thanksgiving and with honour before God and men, all ranks of the Royal Army Medical Corps who have died giving their lives in the service of their King and country."

Next the Anthem, beautifully rendered, by Spohr: "Blest are the departed who in the Lord are sleeping, from henceforth for evermore; they rest from their labours, and their works follow them."

Then came the hymn of Victory, the Lord's Prayer, then a Prayer of Thanksgiving for the life and example of our brothers who, having fought the good fight and finished their course and kept the faith, now rest from their earthly labours. Then the Prayer of Committal: "O God, in Whom all creatures live, in whatsoever world they be, Who by the mouth of Thy Son has taught us that they greatly love who are ready to lay down their lives for others, we commit to the arms of Thy love the souls of those who have fallen in the war. Of Thy mercy, grant O Heavenly Father, that we who serve Thee still on earth may one day with them be partakers of the inheritance of the Saints in light; through Jesus Christ our Lord. Amen."

This was followed by

Commendatory.

"Grant them, O Lord, eternal rest; and let everlasting light shine upon them."

The congregation then prayed for those whom the war has made desolate and broken-hearted, and that we who

**The Worst War of the World**—(*continued*).

were present may be worthy of those who have given their lives for their King and Country.

The Regimental band of H.M. Grenadier Guards, under Capt. A. Williams, M.V.O., rendered the programme of music, which was as follows:—Marche Funèbre et Chant Séraphique, A. Guilmant; Overture, "In Memoriam," Sullivan; Bénédiction, Massanet; Equale, Beethoven. At the end of the service, after the Royal Personages present, the Lord Mayor, the clergy, and the choir had left, the Grenadiers gave Marche Elégiaque, "Gheluvelt," Williams. We had not had the privilege of hearing this march previously, but it is one we should much like to hear again and frequently. It was inspiring and held the hearers throughout, being conducted with great energy by Captain Williams, and beautifully played by the Guards' Regimental Band, which made their rendering of the music, not only a feature of the Service, but excited a keen desire to hear them again. Those members of the congregation, which was a very large one, the Cathedral being full, who did not remain to hear the March Elégiaque, lost something which if they realised and had any love for music, they might not cease to regret to the end of their lives.

Williams' March is in great contrast, of course, to the Dead March in Saul, which the Grenadiers also played with a sympathy and finish that could not be surpassed. We have heard the Last Post and Réveille sounded often in St. Paul's Cathedral and elsewhere, but they were never more effective or striking than at this Service, for the buglers of the Royal Army Medical Corps performed their part of the Service with the maximum of efficiency, remarkable verve and finish. The Dead March was preceded by the Benediction, and one verse (why not two verses?) of "God Save the King" was sung after the Réveille.

We left St. Paul's Cathedral after this service impressed, if possible, more than ever with the meaning of Life and Death. The effects on the spiritual nature of many a man and woman of close and continuous association with the sick, as their human saviours, friends, helpers, and, so often, their confessors. This yields them a quickening realisation of men's and women's spiritual natures, the Fatherly goodness of Almighty God, the strength-giving power of Christ's love for us, as continuously exemplified to the spiritually-minded throughout life, in all circumstances, with the sanctifying influence of the Holy Spirit. Then, too, we may find in men, women, and even little children, the power and consolation that bring peace with joy and courage. Thus arise an abiding feeling and consciousness of how to learn to live the life on earth with an assurance of immortality. So people may become believers endowed with a courage and good cheer which no earthly power can shake, much less destroy.

